# Evaluation of the nano silica and nano waste materials on the corrosion protection of high strength steel embedded in ultra-high performance concrete

**DOI:** 10.1038/s41598-021-82322-0

**Published:** 2021-01-28

**Authors:** Sahar A. Mostafa, Mohamed M. EL-Deeb, Ahmed A. Farghali, A. Serag Faried

**Affiliations:** 1 Department of Civil Engineering, Faculty of Engineering, Beni-Suef University, Beni- Suef, 62511 Egypt; 2 Applied Electrochemistry Laboratory, Chemistry Department, Faculty of Science, Beni-Suef University, Beni-Suef, 62511 Egypt; 3 Materials Science and Nanotechnology Department, Faculty of Postgraduate Studies for Advanced Sciences, Beni-Suef University, Beni-Suef, 62511 Egypt; 4 Department of Civil Engineering, Faculty of Engineering, Fayoum University, Fayoum, Egypt

**Keywords:** Engineering, Materials science, Nanoscience and technology

## Abstract

Corrosion resistance of high strength steel (HHS) embedded in ultra-high performance concrete (UHPC) immersed in 3.5% NaCl solution is evaluated in the absence and presence of nano silica (NS), nano glass waste (NGW), nano rice husk ash (NRHA) and nano metakaolin (NMK) using open circuit potential, potentiodynamic polarization and electrochemical impedance spectroscopy (EIS) under normal and accelerated conditions. Data showed that the corrosion rate in the accelerated conditions is higher compared by the normal conditions due to the increasing in the rate of both anodic and cathodic reactions in the presence of anodic current. On the other hand, the presence of the studied nano materials decreases both the anodic and cathodic overpotentials, and shifts both the open circuit potential (E_ocp_) and corrosion potential (E_corr_) of HSS to more noble values, as well as decreases the values of the corrosion current densities (I_corr_) in both normal and accelerated conditions. Furthermore, EIS analysis illustrates that the presence of these materials enhances both the concrete bulk resistance and the charge transfer resistance at HSS/UHPC interface, which retards the flow of the electrons between the anodic and cathodic sites, thus impeding the propagation of the corrosion process. The inhibitory effect of the studied nano materials for the corrosion of HSS is interpreted on the basis of the change in the microstructure and the compressive strength of the UHPC.

## Introduction

Concrete is considered as the backbone of the construction industry all over the world from an economic point of view. However, its high brittleness, relatively low tensile-to-compressive strength and insufficient flowability as well as exposer to the aggressive marine environment causes a global concern over its lifespan^1,2^. Therefore, ultra-high performance concrete (UHPC) is considered as a new generation of cementitious composite that be recently used in the construction infrastructures technology. The advantages of the UHPC compared to ordinary concrete are concerned to its extraordinary properties such as strength, durability, ductility, very low water/binder ratio and its highly resistance to the environmental conditions^3–6^. UHPC has employed several projects all over the world and has gained interests in complicated applications such architectural features, bridges, roads, rehabilitation, repair and vertical components; such as utilities towers for gas and oil industry applications, windmills towers, hydraulic structures in different countries^7^.

Nano-technology have revealed that, nano-sized particles with high surface area to volume ratio, that provides the potential for tremendous chemical reactivity to achieve the current challenge to get durable concrete with less cement within reasonable cost. Due to the fact that glass waste is not biodegradable, landfills do not offer an ecological solution. A potential solution providing a sustainable, ecological and economic solution are needed. Investigations proved that a particle size less than 75 µm has a pozzolanic reactivity and positive contribution on the micro-structural properties^8^. The pozzolanic nature of rice husk ash due to high silica content makes it a valuable supplementary cementitious material that reduces the land-filling costs, as well as provides a cleaner sustainable environmental solution in saving energy and reducing carbon dioxide generation by cement consumption^9^

Corrosion of steel reinforcing steel bar (rebar) in concrete is one of the most important and frequent factor in long-term civil infrastructure failures around the world^10^. Chloride-induced corrosion of rebar is considered as a main reason for steel corrosion in civil infrastructure, that be occurred due to attacks of chloride ions from marine and chloride contaminated environments^11,12^. Numerous materials can be used to improve the microstructures of UHPC, that can decrease the permeability and diffusibility of the chemicals inducing chlorides to the rebar^13,14^, other waste materials are used to improve the shielding effect of the concrete to gamma radiation^15^. Maddalena et. al.^16^, synthesized the relative simplicity, speed, comparative low cost of C–S–H to improve the mechanical and microstructure in the construction industry. UHPC with 1% nano metaclay enhanced the corrosion resistance for embedded steel^17^. The presence of nano silica (NS) in UHPC, effectively delayed the initiation of the corrosion of steel bars. LPR and Tafel results ensured that NS increased the polarization resistance of embedded bars and lowered corrosion rate^14^. Accelerated corrosion test has the most efficiency technique to measure the corrosion resistance. Some studies have revealed that great differences exist between accelerated corrosion and natural corrosion on corrosion products, distribution of corrosion penetration and developing of expansive cracks^18–20^ Corrosion resistance of high performance concrete (HPC), ultra-high performance concrete (UHPC) and UHPC incoroprating nano silica (UHPC-ns) was evaluated^14^. Results indicated that the time nedded for the first crack was 3600 min, 7000 min, and 8800 min for HPC, UHPC and UHPC-ns respectively, indicating that the corrosion resistance of UHPC-ns is higher than that of UHPC and HPC. Corrosion rate of UHPC-ns was the lowest rate 0.1 mm/year when compared to 0.35 mm/year and 0.17 mm/year for HPC and UHPC respectively. Higher polarization due to nano silica ensures the enhancement of corrosion resistance also corrosion potenial dropped in all cases with the similarity in case of HPC and UHPC but E_corr_of UHPC-ns dropped from 0.511 V to 0.58 V with asignificat values due to nano silica effect in improving the concrete microstructure, strength and durability which plays avital role in lowering the diffusion of chlorides in the concrete specimens^21^. Nano titinium and nano CaCO_3_ in presence of sodium nitrate achieved noticable improvemrnt indurability, strenght, carbonaton and corrosion resistance of green concrete^1^. The inclusion of 1% nano matakaolin enhances the mechanical properties and microstructure of UHPC^22,23^. Tawfik et al.^24^, studied the corrosion rate of reinforcing steel in high-performance concrete using nano additives. They found that the corrosion potentials of the rebar were shifted to more positive values, as well as the corrosion current densities were decreased in the presence of the nano additives compared to control sample.

Aim of our research is to evaluate the effect of nano silica and three types of nano waste materials; nano glass waste (NGW), nano rice husk ash (NRHA) and nano metakaolin (NMK) on the corrosion resistance of HSS embedded in UHPC immersed in 3.5% NaCl solution in normal and accelerated conditions using open circuit potential, Potentiodynamic polarization and electrochemical impedance spectroscopy techniques.

## Experimental

### Materials

Ordinary Portland Cement (type I, 52.5 grade) supplied from Misr Beni Suef company (Beni Suef city, Egypt) according to ASTM C150^25^ is used for casting the concrete specimens. Silica fumes is supplied from Sika Egypt Company with specific surface area of 16.8 × 103 m^2^/Kg and 2.15 specific gravity, is used as mineral admixture based on the mass of the cement. The aggregate consisted of crushed dolomite from Ataqa mountain quarry with a maximum size of 6.3 mm and specific density of 2.65 as a coarse aggregate, while natural sand with fineness modulus of 2.94 mm as a fine aggregate. The selection of the aggregates is in agreements with ASTM C33^26^. Coarse aggregate is used in saturated surface dry conditions, while fine aggregate is washed, dried and used in dry conditions. Additional amount of water equal to absorption of sand is added to the effective water (w/b). Polycarboxylates water reducer (Sika Viscocrete 3425) with density of 1.08 t/m^3^ is used as a superplasticizer with 1.0 – 1.5% based on the mass of the cement. 35 mm hooked-end steel fibers with aspect ratio of 43.75 and tensile strength of 1100 MPa are used to improve the ductility and increase the energy absorption of the ultra-high performance concrete (UHPC).

**Figure 1:**
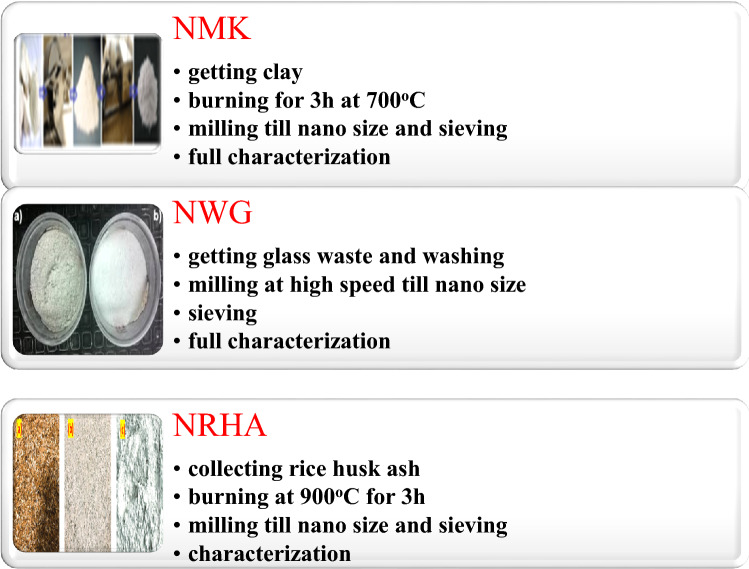
Preparing of nano waste materials.

**Figure 2:**
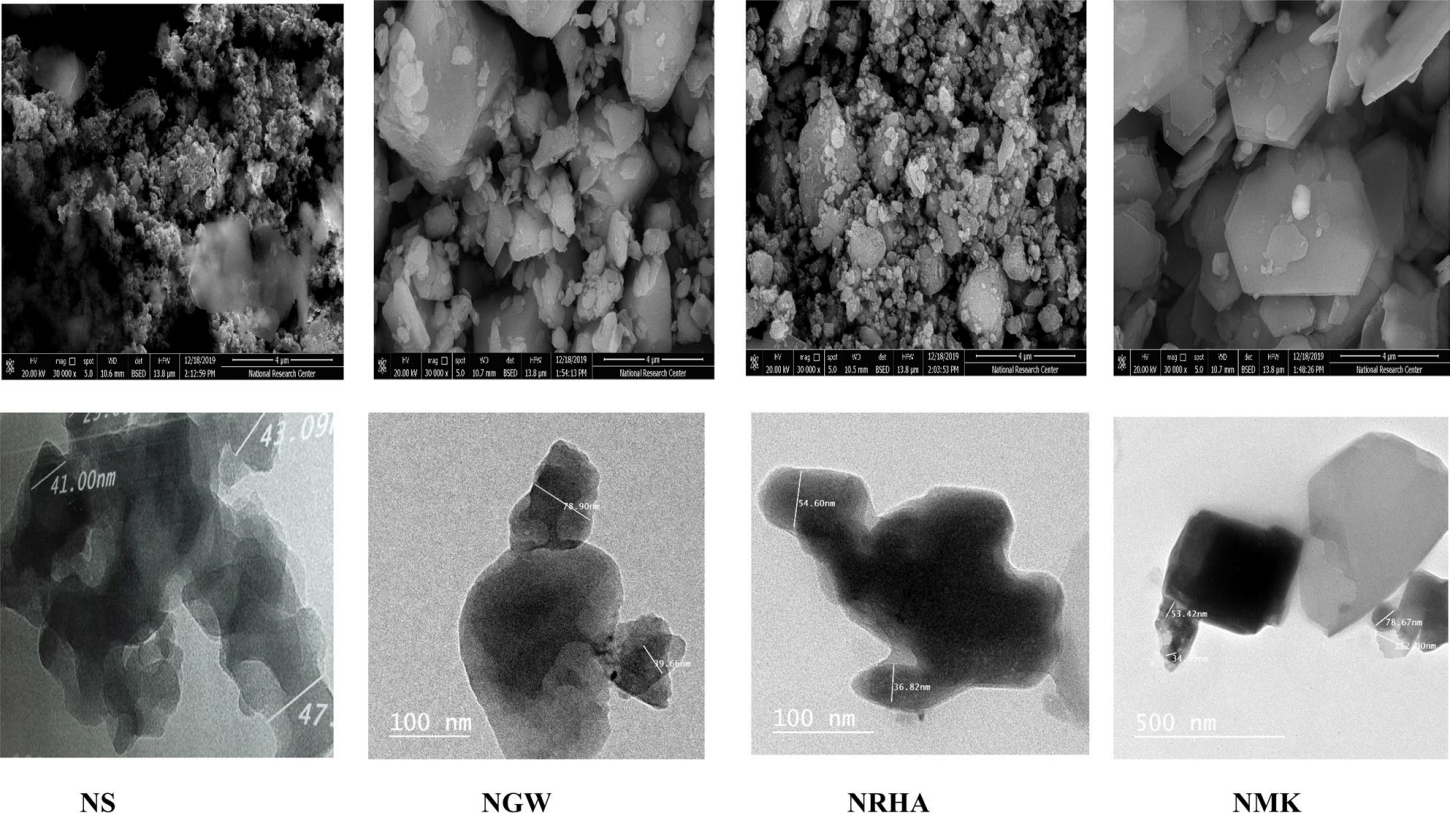
Microstructure of nano silica and nano waste materials.

Nano silica (NS) and three types of nano waste materials; nano glass waste (NGW), nano rice husk ash (NRHA) and nano metakaolin (NMK) are prepared in nano sized scale, in our laboratory as discussed previously^23,27–29^ which schematically represented in Fig. 1, and their chemical composition are tabulated in Table 1, as well as their microstructure is shown in Fig. 2. The mix design for casting the UHPC specimens is based on Maravelaki-Kalaitzaki et al.^30^ and are summarized in Table 2.

**Table 1:** Chemical composition of nano materials.

Items	SiO_2_	Al_2_O_3_	Fe_2_O_3_	CaO	MgO	SO_3_	K_2_O	Na_2_O	Cl	TiO_2_
NS	95.39	0.15	1.11	0.43	0.09	0.05	0.030	1.790	0.71	–
NWG	72.58	0.17	1.11	12.12	2.09	0.19	0.030	11.700	0.01	–
NMK	89.6	0.9	2.0	0.43	2.0	–	4.55	–	–	0.7
NRHA	92	0.23	0.21	1.39	0.36	1.42	2.99	0.2	0.16	–

**Table 2:** Mix proportions of UHPC.

Sample	Cement (kg/m^3^)	SF (kg/m^3^)	Fine aggregate (kg/m^3^)	Coarse aggregate (kg/m^3^)	Water (kg/m^3^)	SP (kg/m^3^)	Steel fiber % (volume fraction)	NWG %	NS %	NMK %	NRHA %	Compressive strength (MPa)
UHPC	900	135	348.75	776.25	186.3	22.5	1	–	–	–	–	114.7
UHPC-NRHA	900	135	348.75	776.25	186.3	22.5	1	–	–	–	1	118.4
UHPC-NMK	900	135	348.75	776.25	186.3	22.5	1	–	–	1	–	119.8
UHPC-NS	900	135	348.75	776.25	186.3	22.5	1	–	1	–	–	130.9
UHPC-NWG	900	135	348.75	776.25	186.3	22.5	1	1	–	–	–	132.1

### HSS rebar

The studied high strength steel (HSS) is produced according to Egyptian Standards as deformed bars (ES 262/2009 Gr), its chemical composition as provided from the manufacturer is shown in Table 3.

**Table 3:** Chemical composition of steel rebar.

Fe	C	Mn	Si	S	P	N	Sn	V	Mo	Cr	Co	CEV
96.948	0.29	1.6	0.55	.040		0.0120	0.56					

### Sample geometry

**Figure 3:**
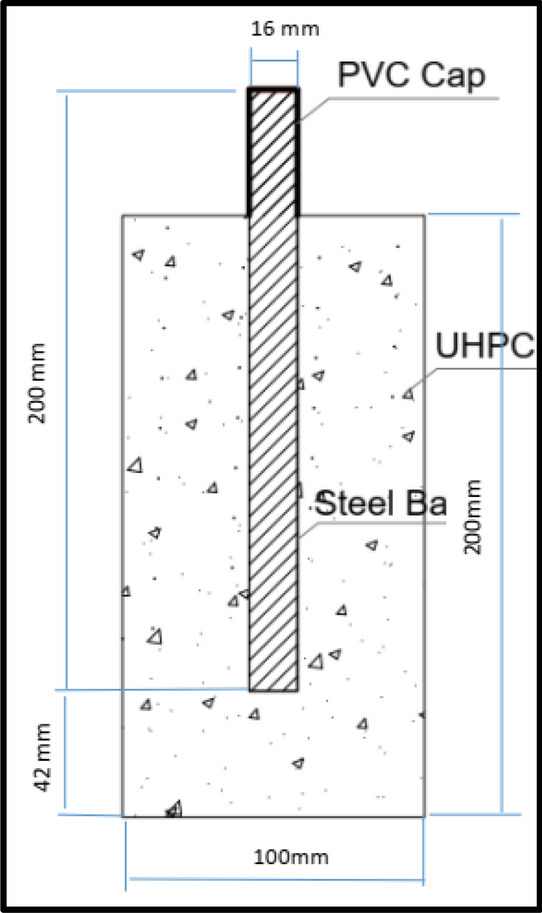
Geometry of the HSS rebar (Autocad 2018).

UHPC cylindrical specimens with 100 mm diameter and 200 mm height are casted for the electrochemical tests. HSS rebar with 16 mm diameter and 200 mm height is embedded in UHPC mix at the middle of the cylindrical specimens at a distance of 42 mm from concrete base as schematically represented in Fig. 3.

### Electrochemical measurements

All the electrochemical measurements are carried out using the Potentiostat / Galvanostat (AUTOLAB PGSTAT 128 N) as previously reported^31^. In short, standard three-electrode cell with HSS rebar working, saturated Ag/AgCl reference and Pt sheet counter electrodes are used, while NOVA 1.11 software is used to records and fits the electrochemical measurements. Potentiodynamic polarization measurements are achieved in the potential range between –100 to + 100 mV vs. E_OCP_ values at 25 ± 2 °C with the scan rate of 1.0 mV s^-1^. Electrochemical impedance spectra at the respective E_OCP_ values are recorded using AC signals of amplitude 5 mV peak to peak in the frequency range of 10 kHz to10 MHz.

### Impressed voltage test

**Figure 4:**
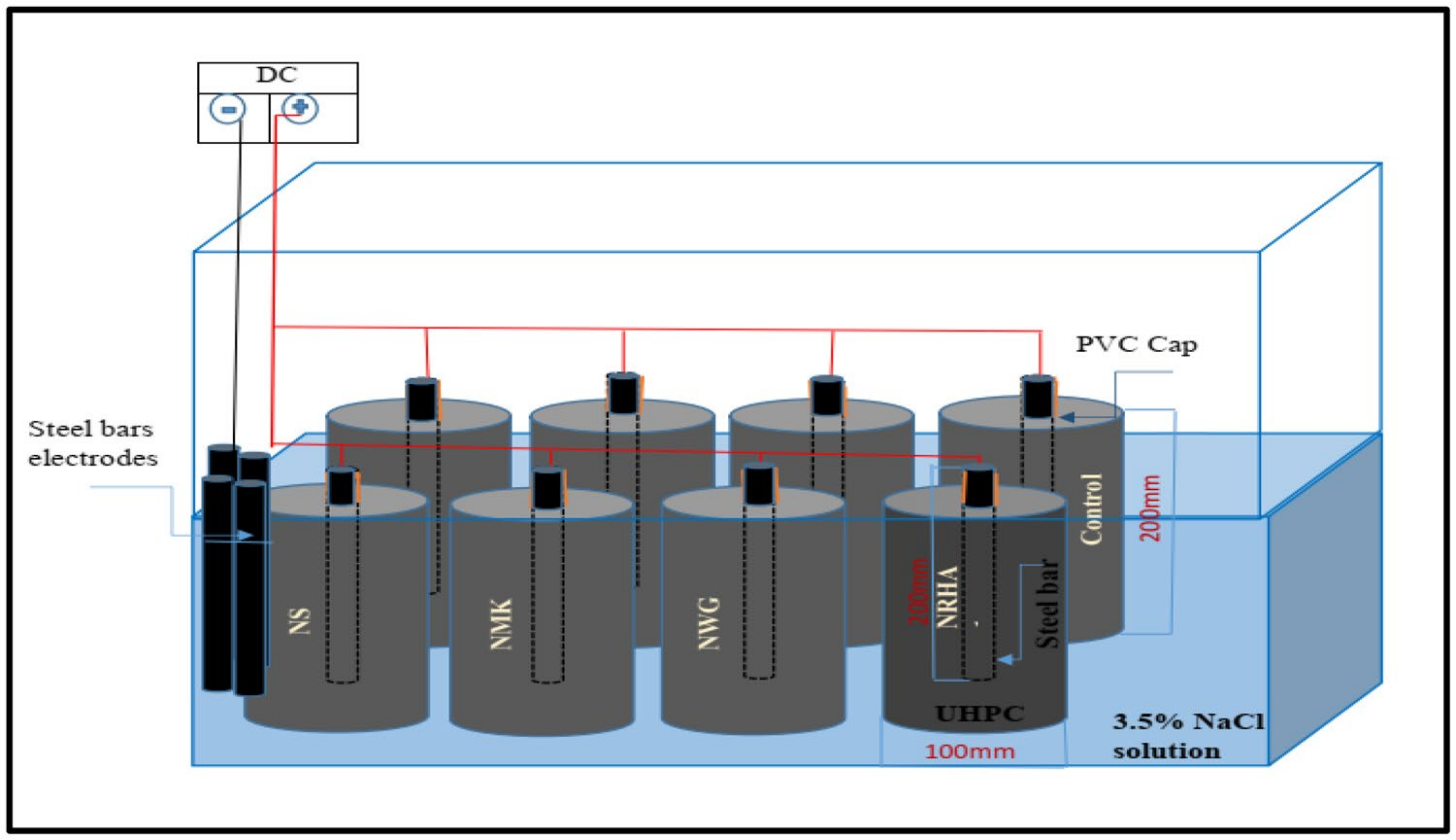
Setup for impressed voltage test (Microsoft office 2013).

The impressed voltage technique is considered as an accelerated technique used to investigate the corrosion resistance of the rebar^32^. The test setup is represented in Fig. 4, and occurred by immersing the specimens in 3.5% NaCl solution. HSS rebar acts as the anode and a cylindrical steel acts as the cathode, while a constant DC potential of 12 V is impressed between the anode and cathode using a DC power supply.

### Microstructure characterization

The microstructures of the prepared materials are examined using a Transmission Electron Microscope (JEOL JEM 2100, Japan) with an acceleration voltage of 200, SEM–EDX analysis is carried out using JSM-6510LA (JEOL, Tokyo, Japan). Crystallinity and the phase identification are analyzed using X-ray diffractometer (PANalytical Empyrean, Netherlands) using CuKa radiation (wavelength λ = 1.54045 Å) at an accelerating voltage 40 kV, current 35 mA.

Raman Spectroscopy RS are illuminated with a 532 nm He–Ne. Laser scanning is carried out by using the syncro mode from 0 to 2000 cm^-1^. Data are performed with WiRE3.3 software (Renishaw, Illinois). BET surface area, pore volume and pore size distribution are determined using a surface area analyzer (TriStar II 3020, Micromeritics, USA).

### Compressive strength test

Concrete cube specimens of size 150 mm × 150 mm × 150 mm are casted and subjected to compressive strength test as per ASTM C39^33^ using a 3000 kN capacity digital compressive testing machine at the age of 28 days.

## Results and discussion

### Open circuit potentials

**Figure 5:**
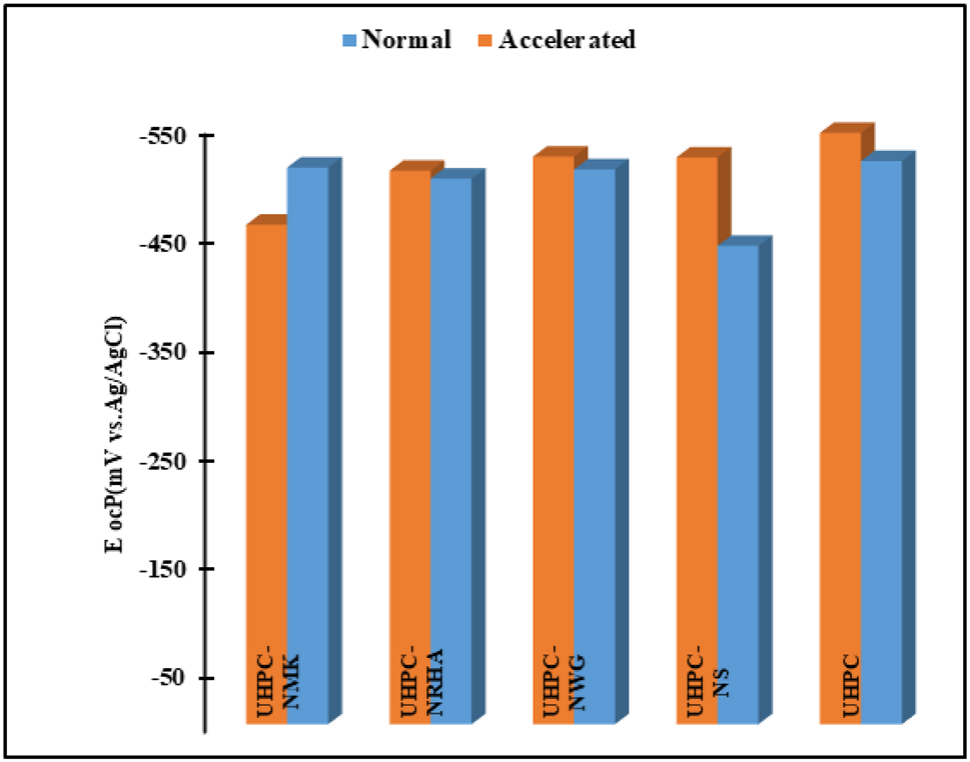
variation of E_ocp_ of HSS embedded in UHPC immersed in 3.5% NaCl in the absence and presence of 1% nano materials at 25 ± 2 °C in both normal and accelerated conditions.

Changes in the open circuit potentials (E_OCP_) values of HSS embedded in UHPC that been immersed for 50 days in 3.5% NaCl solution in the absence and presence of 1% NS, NGW, NRHA and NMK under normal and accelerated conditions at 25 ± 2 °C are shown in Fig. 5 and Table 4. Data clearly shows that, more negative value of Eocp for HSS embedded in the control specimen (UHPC) in accelerating conditions compared by its value in the normal conditions. This results are attributed to the increasing in the rate of both anodic and cathodic reactions in the presence of anodic current, that increases the overall corrosion rate. These reactions can be summarized^11,34^ as:

1. The anodic dissolutions of HSS that release electrons and dissolves as ferrous ions as represents in the following equation:
  1 $$ {\text{Fe}} \to {\text{Fe}}^{2 + } + 2e^{ - } $$
2. These electrons migrate to the cathodic site to reduce O_2_ and/or H_2_O as follows:
  2 $$ {\text{O}}_{2} + 2{\text{H}}_{2} {\text{O}} + 4e^{ - } \to 4{\text{OH}}^{ - } $$
  3 $$ {\text{2H}}_{2} {\text{O}} + 2e^{ - } \to 2{\text{OH}}^{ - } + {\text{H}}_{2} $$
3. Then Fe^2+^ contentious to react as follows to form rust:
  4 $$ {\text{Fe}}^{ + } + 2{\text{OH}}^{ - } \to {\text{Fe}}({\text{OH}})_{2} $$
  5 $$ {\text{4Fe(OH)}}_{2} + 2{\text{H}}_{2} {\text{O}} + {\text{O}}_{2} \to 4{\text{Fe}}({\text{OH}})_{3} $$
  6 $$ {\text{6Fe(OH)}}_{2} + {\text{O}}_{2} \to 6{\text{H}}_{2} {\text{O}} + 2{\text{Fe}}_{3} {\text{O}}_{4} $$

**Table 4:** Electrochemical kinetic parameters of HSS embedded in UHPC immersed in 3.5% NaCl in the absence and presence of 1% nano materials.

Sample	Accelerated condition					Normal condition				
	E_ocp_ mV (vs. Ag/AgCl)	E_corr_ mV (vs. Ag/AgCl)	I_corr_ (µA.cm^-2)^	R_p_ (Ω)	C_Rate_ mm/year	E_ocp_ mV (vs. Ag/AgCl)	E_corr_ mV (vs. Ag/AgCl)	I_corr_ µA.cm^-2^	R_p_ (Ω)	C_Rate_ mm/year
UHPC	− 544.2	-547.2	39.14	415.8	0.454	-518.3	− 520.4	31.72	1060.6	0.369
UHPC-NS	− 521.6	-505.8	20.39	808.9	0.237	-440.2	− 448.4	07.63	2701.3	0.088
UHPC-NRHA	− 509.3	-524.2	16.96	541.6	0.197	-504.1	− 513.4	14.49	1682.3	0.168
UHPC-NGW	− 522.1	-468.7	12.41	2324.0	0.144	-514.9	− 520.9	12.68	1984.3	0.147
UHPC-NMK	− 459.9	-522.3	11.92	1217.2	0.138	-512.3	− 519.1	25.36	1022.7	0.327

**Figure 6:**
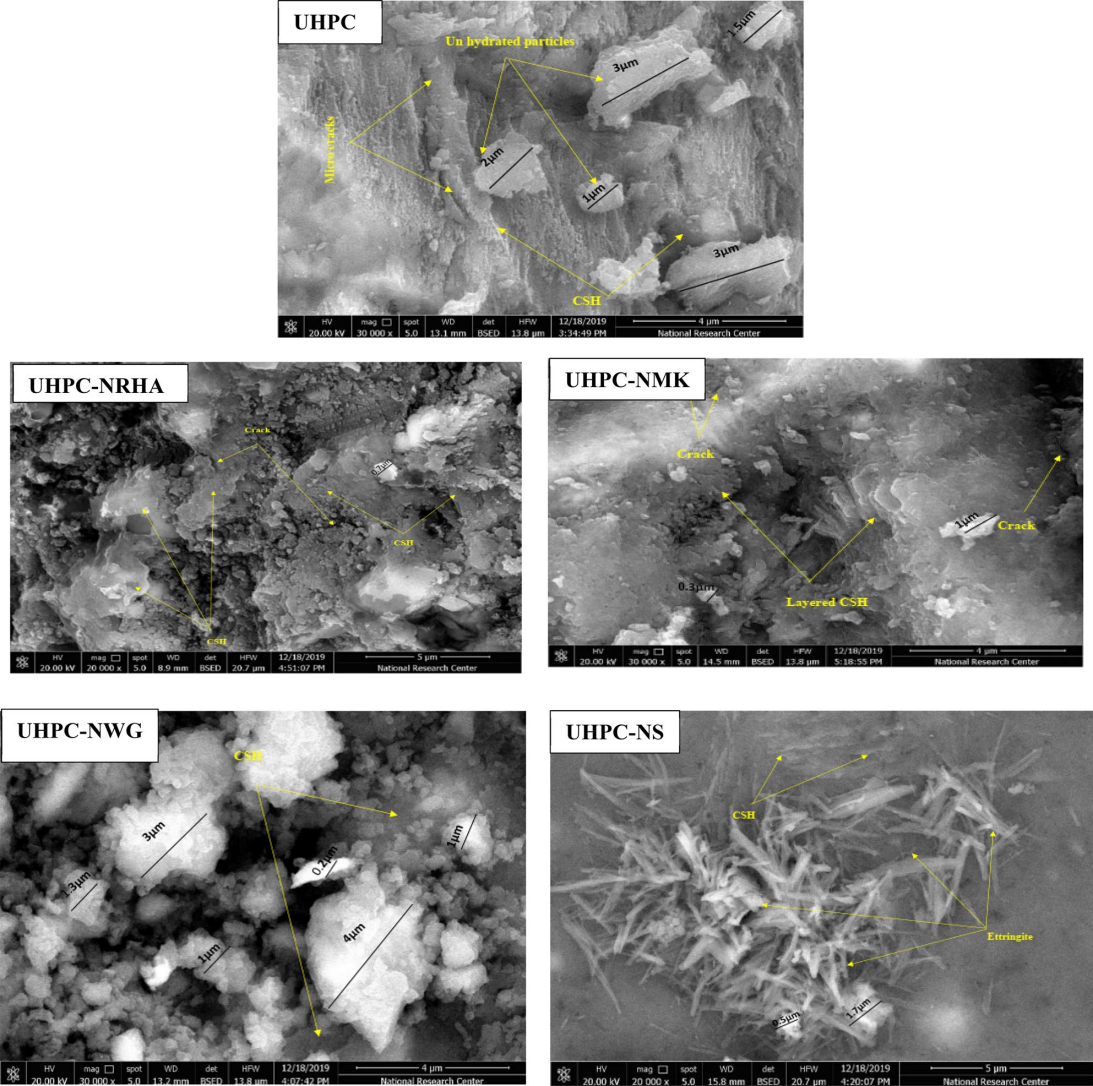
SEM micrographs of UHPC in the absence and presence of the studied nano materials.

Also, we can conclude from that figure that, the incorporating of the studied nano materials in UHPC, enhances the corrosion resistance of HSS in both normal and accelerating conditions. This behaviour can be explained as a result of the synergistic effect of these nano materials on the microstructure and mechanical properties of UHPC as summarized in Fig. 6 and Table 2. It is found that, the SEM micrograph of UHPC shows denser microstructure with no evidence of micro cracks propagation in the presence of the studied nano materials compared to UHPC control specimen. Specific surface area of UHPC and its composite with the studied nano materials are studied by BET multi-point method and BJH method. Results of the specific surface area, total pore volume and pore size analysis are tabulated in Table 5. It can be seen from the Table 5 that the incorporation of the nano materials with the UHPC shows microporous structure (pore size ˂ 10 nm) with small both specific surface area and total pore volume, compared to UHPC which follows the following order: UHPC ˃ UHPC-NMK ˃ UHPC-NRHA ˃ UHPC-NWG ˃ UHPC-NS. This observation can be interpreted to the filling effect of these materials on both the pores and the micro cracks of UHPC as well as their effect on the pozzolanic reaction that enhances the compactness structure of the UHPC composite. On the other hand, the order of the dense structure of the UHPC composites are in a good agreement with their measured compressive strength as indicated from Table 2. This improvement in the dense pore structure and the compressive strength of the UHPC composites, enhances the inhibitory effect of the diffusion and penetration of O_2_, H_2_O and Cl^-^ to HSS/UHPC interface. Therefore, reduces the rate of the cathodic reaction and thereby the overall corrosion rate. On the other hand, the presence of these nano materials increases the electrical resistivity of UHPC that retards the flow of electrons between the anodic and cathodic sites, thus impeding the propagation of the corrosion process^10^. This data is in a good agreement with our EIS measurements.

**Table 5:** Surface area analysis of UHPC nano composites.

Samples	S _BET_ (m^2^/g)	Total pore volume (cc/g)	Pore size (nm)
UHPC	168.9	0.32	3.8
UHPC-NMK	140	0.75	1.07
UHPC-NRHA	17.46	0.045	4.6
UHPC-NGW	15.2	0.042	5.5
UHPC-NS	7.3	0.037	1.02

### Potentiodynamic polarization measurements

**Figure 7:**
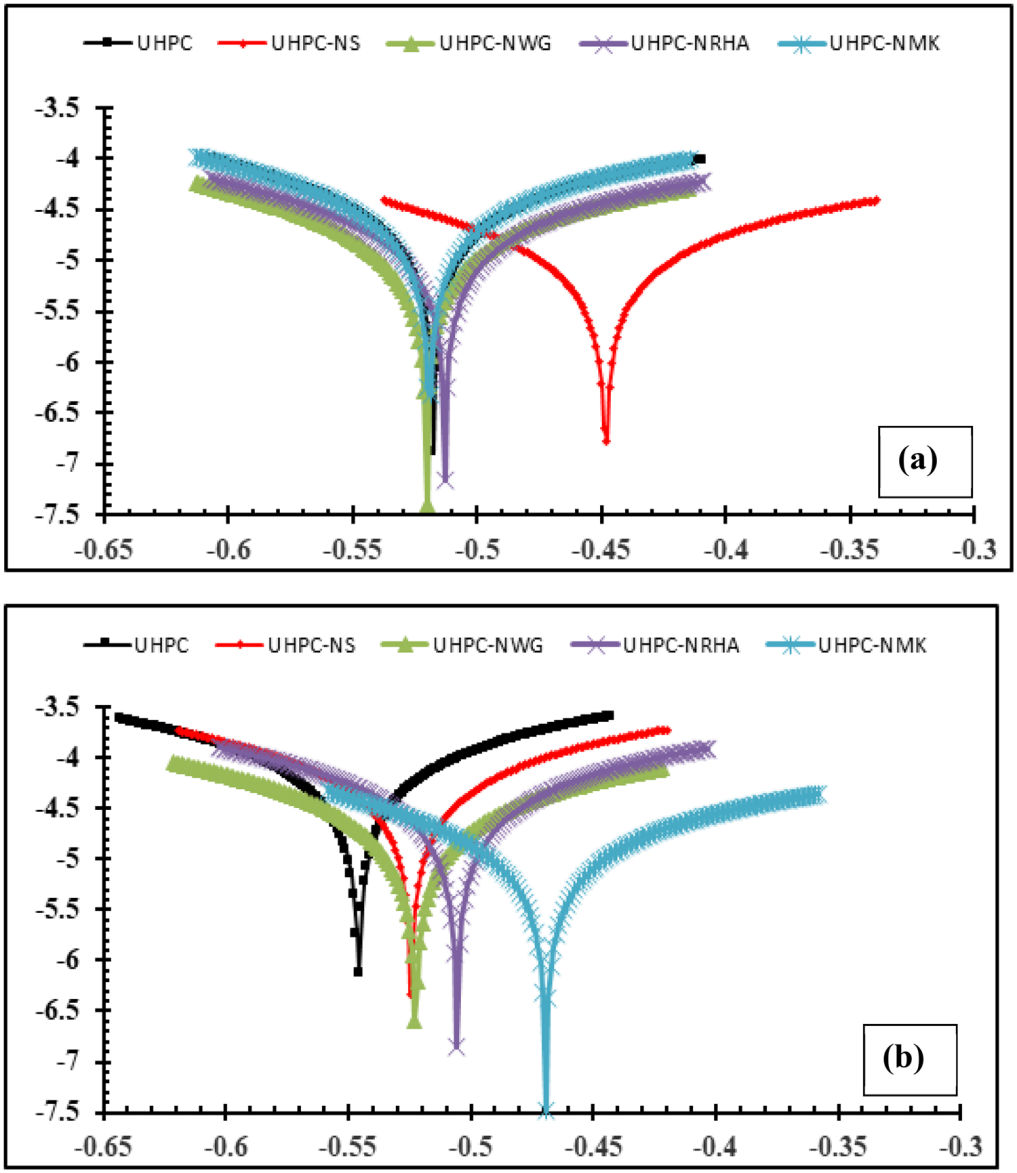
Potentiodynamic polarization plots of HSS embedded in UHPC immersed in 3.5% NaCl in the absence and presence of 1% nano materials at 25 ± 2 °C, with scan rate of 1.0 mV/s in (**a**) normal and, (**b**) accelerated conditions.

Potentiodynamic polarization measurements are conducted for HSS rebar embedded in UHPC after immersion in 3.5% NaCl solution for 50 days, by changing the electrode potential automatically ± 100 mV against the E_OCP_ with scan rate of 1.0 mV/s. Figure 7 represents the anodic and cathodic polarization curves of HSS embedded in UHPC in the absence and presence of 1% NS, NGW, NRHA and NMK, under normal and accelerated conditions at 25 ± 2 °C, while there electro-chemical kinetic parameters are tabulated in Table 4. The results imply that the presence of uniform corrosion without any sign for pitting corrosion within the studied polarization range for all specimens under normal and accelerated conditions. Moreover, the corrosion potential (E_corr_) of the control specimens (UHPC) is found to be -547.2 and -520.4 mV (vs. Ag/AgCl) while the corrosion current density (I_corr_) of 39.14 and 31.72 µA.cm^-2^ in the accelerated and normal conditions respectively. On the other hand, the calculated corrosion rate (C_Rate_) for HSS in normal condition is 0.369 mm/year compared to 0.454 mm/year in case of accelerated condition. The higher both I_Corr_ and C_Rate_ with more negative shift in the E_corr_ for HSS in accelerated condition, compared to normal condition can be attributed to the higher both anodic and cathodic reactions in the presence of anodic current as discussed before.

**Figure 8:**
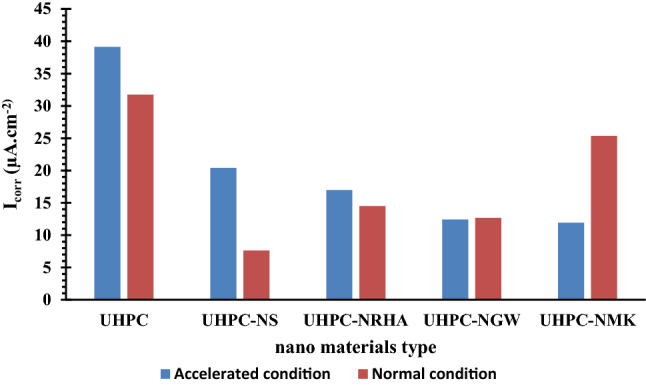
Variation of I_corr_ of HSS embedded in UHPC immersed in 3.5% NaCl in the absence and presence of 1% nano materials at 25 ± 2 °C in both normal and accelerated conditions.

**Figure 9:**
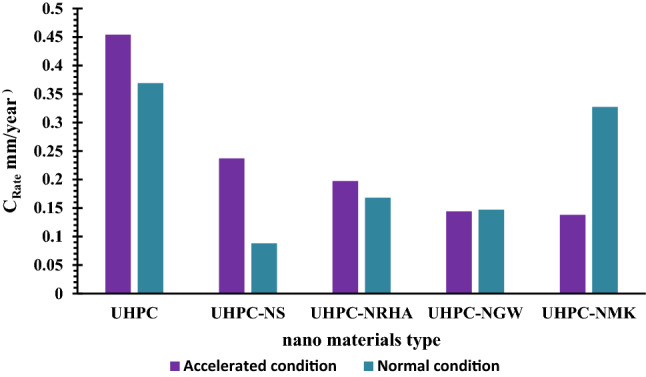
Variation of C_Rate_ of HSS embedded in UHPC immersed in 3.5% NaCl in the absence and presence of 1% nano materials at 25 ± 2 °C in both normal and accelerated conditions.

**Figure 10:**
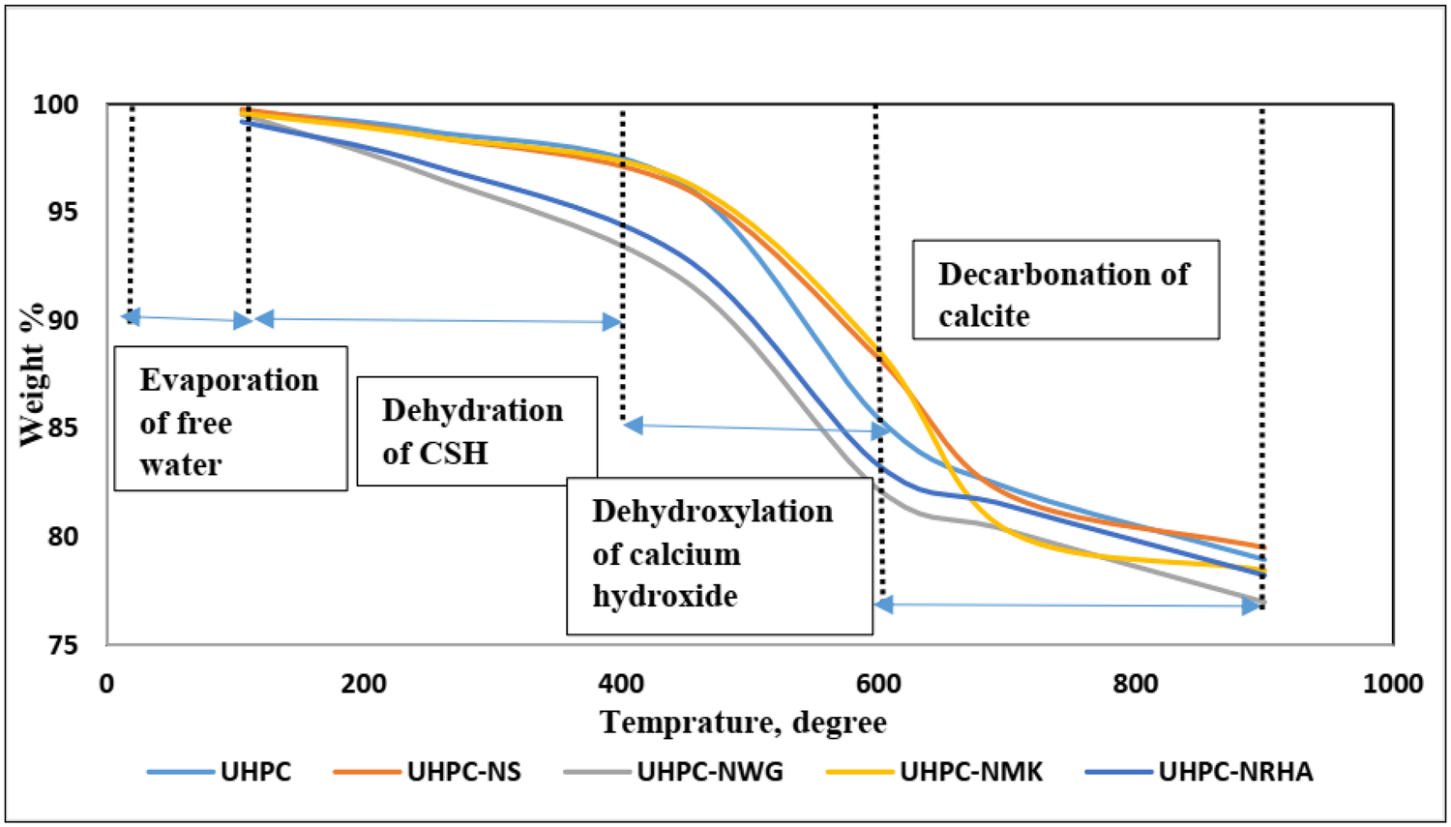
TGA thermograms of UHPC in the absence and presence of the studied nano materials.

**Figure 11:**
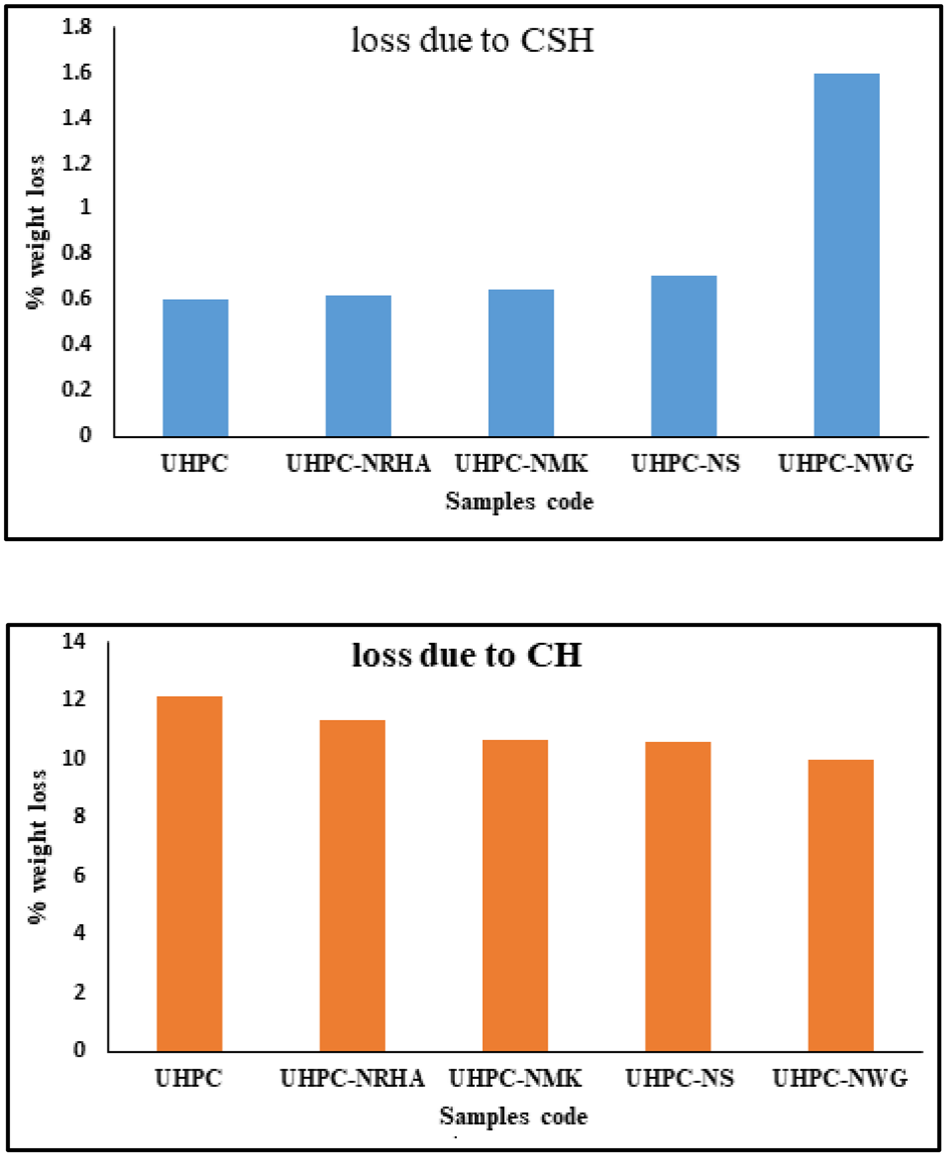
Variation of CSH & CH % as a function of the studied nano materials.

**Figure 12:**
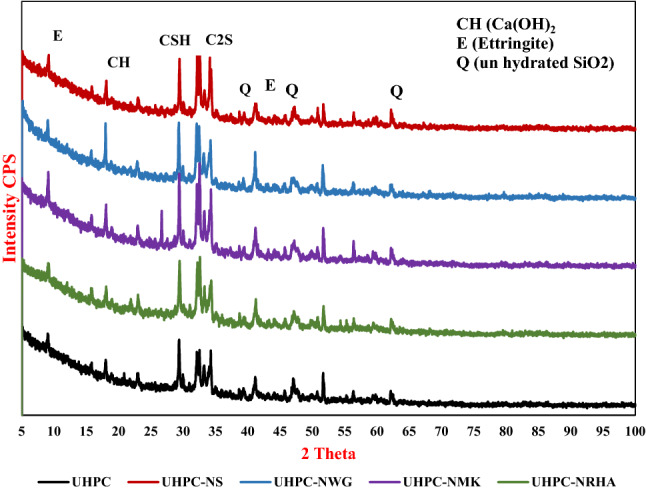
XRD spectra the studied nano materials composites.

**Figure 13:**
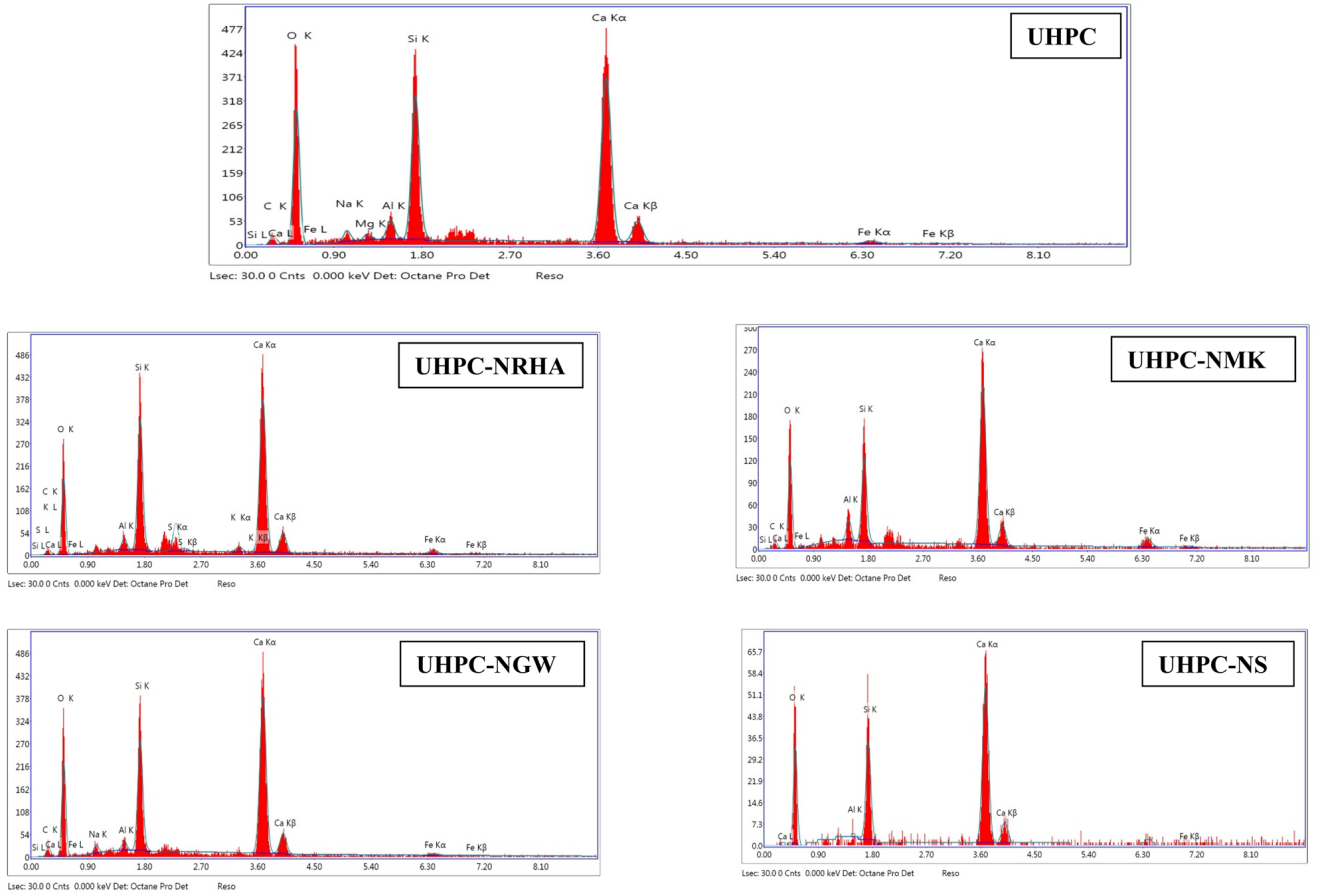
EDX analysis of UHPC in the absence and presence of the studied nano materials.

**Figure 14:**
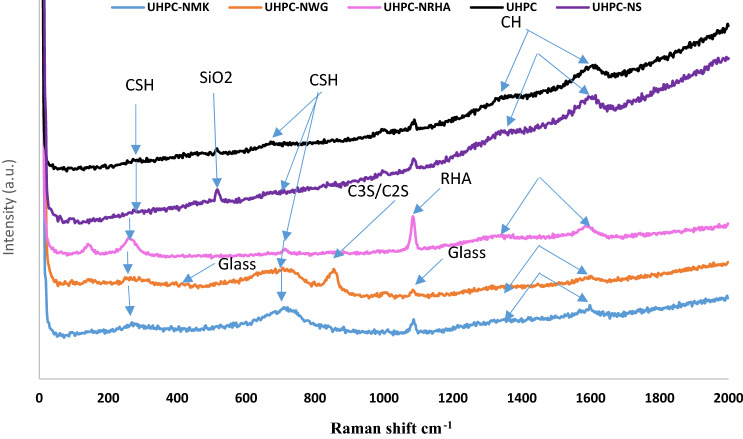
Raman spectroscopy for UHPC in the absence and presence of nano materials.

Addition of 1% nano materials to the UHPC, decreases both the anodic and cathodic polarization of HSS, and shifts its E_Corr_ to more noble values, as well as decreases both I_Corr_ and C_Rate_ compared to control specimen in both conditions. Figure 8 shows the variation of I_corr_ of HSS as a function in the nano material types, as indicated from the figure, the values of I_corr_ are declining from 39.14 µA.cm^-2^ for UHPC control specimen in the accelerated conditions to 20.39, 16.69, 12.41 and 11.92 µA.cm^-2^ for UHPC-NRHA, UHPC-NS, UHPC-NMK and UHPC-NGW respectively. Also, in the normal condition the values of I_Corr_ are decreased follows the following order: UHPC ˃ UHPC-NMK ˃ UHPC-NRHA ˃ UHPC-NGW ˃ UHPC-NS. We can conclude from the above results, the incorporating of the studied nano materials during the UHPC mix, increases the corrosion resistance of HSS rebar embedded in UHPC. These results are in a good agreement with the values of the calculated corrosion rate that graphically represented in Fig. 9. This effect is explained on the basis of the dual effect of these nanomaterials which, (1) Effectively filling the micro pores that reduces the permeability and improves the UHPC' microstructure and thus its corrosion resistance as discussed before. (2) Accelerates the rate of the hydration reaction which, acts as the nucleation sites for the formation of the hydration products; calcium hydroxide (CH) and calcium silicate hydrate (CSH). On the other hand, the presence of active SiO_2_ in these nano materials, enhances the CSH/CH ratio, which increases the gel pore in UHPC microstructure, that leads to improving its mechanical properties; as observed in its compressive strength (c.f. Table 2), and thereby its corrosion resistance. Figure 10 shows TGA thermograms of UHPC in the absence and presence of the studied nano materials, while the variation of CH and CSH %, that been calculated from TGA data based on Taylor's equation^35^, are graphically represented in Fig. 11. Results show that the CSH% increases with the following order: UHPC ˂ UHPC-NRHA ˂ UHPC-NMK ˂ UHPC-NS ˂ UHPC-NGW. This order is well agreement with our results of the values of both HSS corrosion resistance and UHPC compressive strength. Also, XRD results clarify the presence of diffraction peaks corresponds to CSH at 2θ values of 26.8°, 33.1°, 40.9°, 58.8°, 70.8° and 81.3° (JCPDS 00-029-0373)^1^ with high intensity in the UHPC incorporating with nano materials compared to UHPC control specimen as shown in Fig. 12. This can be interpreted to the increasing in the rate of the hydration reaction and thereby the hydration products, as a result of the incorporation of the nano materials to UHPC. This finding is agreed well with the higher Ca/Si molar ratio which is calculated using EDX analysis in the presence of the studied nano materials as shown in Fig. 13. Moreover, Fig. 14 shows Raman patterns for UHPC and its composites with nano materials to assign the characteristic Raman shifts, as a reference to identify each individual components in the hydration process. It is observed that the characteristic peak of silica fume is observed as a strong and unique at 517 cm^-1^, with different intensity due to pozzolanic reactivity and silica reinforced nano materials. Its highest intensity is obtained at UHPC-NS composite, due to the overlapping between nano silica and silica fume. In addition, silica fume can react with calcium hydroxide (CH) in the presence of water during hydration process, resulting calcium silicate hydrate (CSH) which observed as strong and highest intensity at 342 and 665.58–711.4 cm^-1^ for UHPC, at 339.7 and 638–684.51 cm^-1^ for UHPC-NS, at 340.3 and 647– 671 cm^-1^for UHPC-NMK, at 337.4 and 647.2–671 cm^-1^ for UHPC-NWG and at 337 and.

641.8–709 cm^-1^ for UHPC-NRHA. Also, calcium hydroxide (CS) peaks decreased in the presence of nano materials, that can be explained to the effect of these nano materials on the rate of the pozzolanic reaction^36^. Also, the highly pozzolanic reaction and phase transfer in UHPC nanocomposites compared to UHPC is observed from the higher CSH/CH phase ratio in case of nanocomposites. This finding is in a good agreement with the previous results which is calculated from the TGA thermograms.

### Electrochemical impedance spectroscopy

**Figure 15:**
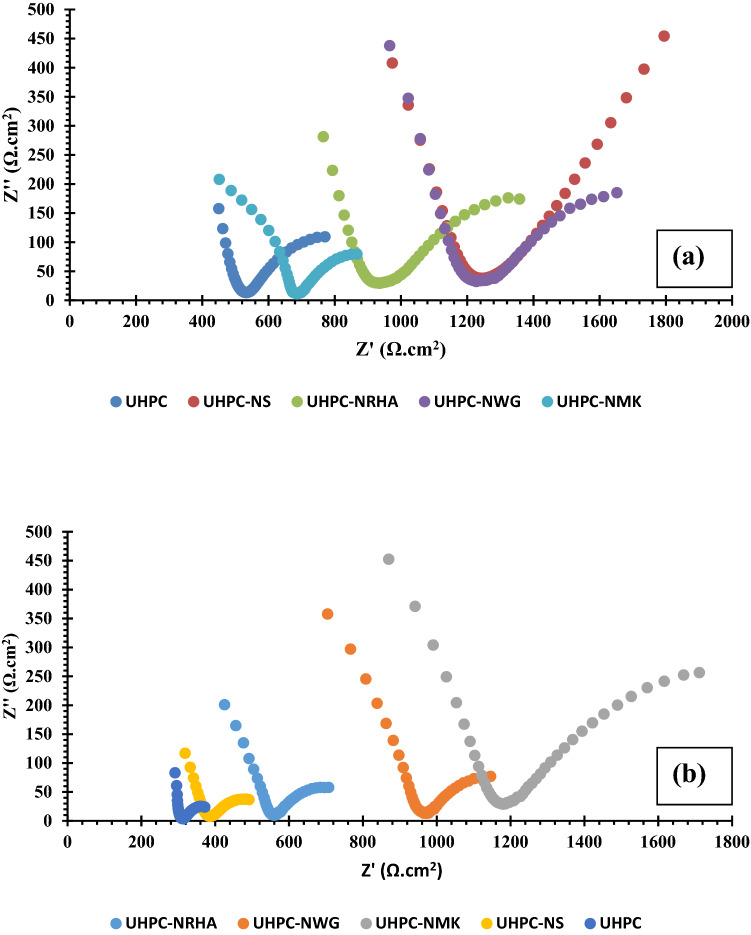
Nyquist plots of HSS embedded in UHPC immersed in 3.5% NaCl in the absence and presence of 1% nano materials at 25 ± 2 °C at E_OCP_ in (**a**) normal and, (**b**) accelerated conditions.

EIS is a non-destructive useful technique which provides a detailed characteristic about Metal/solution and Metal/concrete interface^37–39^. Figure 15 represents the Neyquist plots of HSS embedded in UHPC in the absence and presence of NS, NWG, NRHS and NMK in both normal and accelerated conditions at Eocp over the frequency range of 100 kHz to 10 MHz. Data point out that the presence of two consecutive capacitive semicircles, that have been interpreted to the dielectric properties of the concrete bulk in the high frequency region and to the steel/concrete interface at low frequency region^40^. The diameter of these capacitive loops increases without any change in the shapes of the Nyquist plots in the presence of the studied nano materials, suggesting the mechanism of the corrosion resistance in the absence and presence of these materials is not changed^39^. The increase in the diameters of both two capacitive loops that equivalent to the concrete resistance and charge transfer resistance can be explained to the effect of these materials on the microstructure of the UHPC as discussed before. Denser microstructure due to the effective filling of pore by these nano materials, retards the diffusion of the corrosive ions and H_2_O/O_2_ molecules through the UHPC bulk to the HSS/UHPC interface. Therefore, decreases both the anodic dissolution of HSS and the cathodic reduction of O_2_ and/or H_2_O. This finding indicates the corrosion resistance has been enhanced and the increase in the capacitive loops diameter follows the following order: UHPC ˂ UHPC-NMK ~ UHPC-NRHA ˂ UHPC-NWG ~ UHPC-NS in the normal conditions and in the accelerated conditions as per: UHPC ˂ UHPC-NS ˂ UHPC-NRHA ˂ UHPC-NGW ˂ UHPC-NMK.

**Figure 16:**
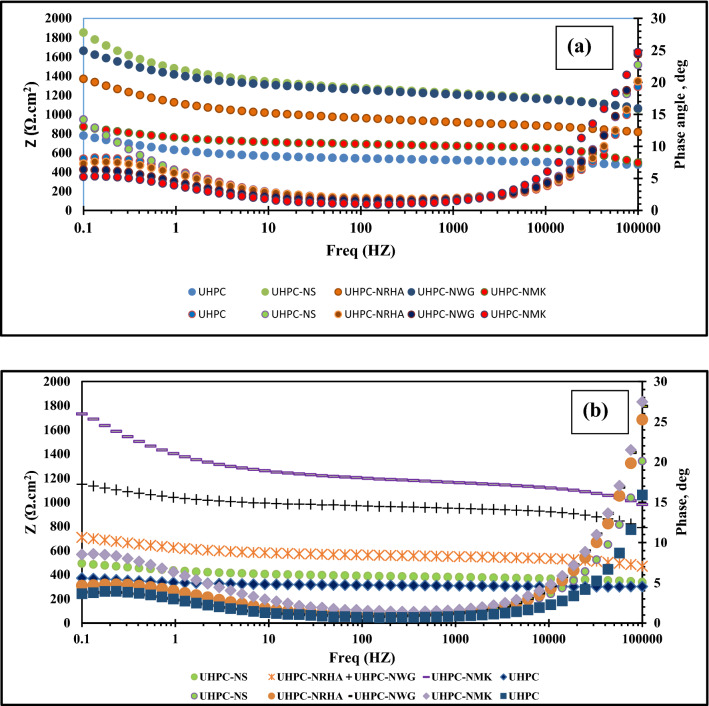
Bode plots of HSS embedded in UHPC immersed in 3.5% NaCl in the absence and presence of 1% nano materials at 25 ± 2 °C at E_OCP_ in (**a**) normal and, (**b**) accelerated conditions.

Figure 16 illustrates the Bode plots obtained for HSS embedded in UHPC, data shows a broad time constant joined by two time constants at high and low frequency regions that attributed to dielectric properties for the concrete and the HSS/UHPC interface as discussed before. The conjunction of the two time constants indicating that the frequency responses of the two time constants are similar^41^. On the other hand, gradual increase of both the area under the curve and phase angle in the presence of these materials compared to the control specimen means the improvement of the UHPC' resistance against the diffusion of the corrosive ions, and thus increases the corrosion resistance for the HSS^42–44^.

**Figure 17:**
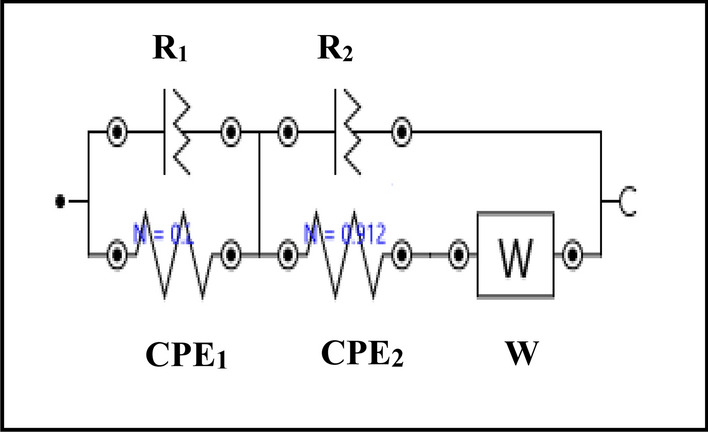
Fitted electrochemical equivalent circuit model using NOVA 1.11 software.

The experimental data are fitted to an electrochemical equivalent circuit based on the Boukamp model^45^, and its model is represented in Fig. 17. It can be seen that the Neyquist plots show [RQ/Q(RW)] equivalent circuit with two-time constant that agreed with the electrochemical equivalent circuit that fitted steel/ concrete systems^11,46–50^. In this model, R_1_ and R_2_ denote the concrete and charge transfer resistances respectively, while the dielectric properties of the concrete bulk and HSS/UHPC interface are represented by CPE_1_ and CPE_2_. Moreover, the diffusion of the charged species is represented by Warburg impedance (W)^11^. The replacement of pure double layer capacitance (C_dl_) with both constant phase element (CPE) and phase shift (N) aims to the compensation of the depression of semicircles in the Nyquist plots that related to the surface roughness. The calculated values of R2 and CPE2 of HSS embedded in UHPC corresponding to the charge transfer reaction through the corrosion process shows a change in the structure of the HSS/UHPC interface in both normal and accelerating conditions for nano composite samples compared to UHPC. Data clarify that, increasing the values of the charge transfer resistance (R2) and decreasing the values of the constant phase elements (CPE2) are observed in the presence of nano materials as per the following order: UHPC (R2 = 502 Ω, CPE2 = 3.7 × 10^3^ µMho) , UHPC-NMK (R2 = 750 Ω, CPE2 = 3.4 × 10^3^ µMho), UHPC-NRHA (R2 = 866 Ω, CPE2 = 3.1 × 10^3^ µMho), UHPC-NWG (R2 = 1.25 kΩ, CPE2 = 1.75 × 10^3^ µMho), UHPC-NS (R2 = 2.33 kΩ, CPE2 = 0.8 × 10^3^ µMho) in the normal conditions and in the accelerated conditions as follows: UHPC (R2 = 231 Ω, CPE2 = 4.1 × 10^3^ µMho), UHPC-NS (R2 = 362 Ω, CPE2 = 3.4 × 10^3^ µMho), UHPC-NRHA (R2 = 399 Ω, CPE2 = 2.9 × 10^3^ µMho), UHPC-NGW (R2 = 1.68 kΩ, CPE2 = 2.1 × 10^3^ µMho), UHPC-NMK (R2 = 2.23 kΩ, CPE2 = 0.7 × 10^3^ µMho). This behaviour can be explained to the effect of the studied nano materials on the UHPC microstructure which retards both the diffusion and the penetration rates of H_2_O molecules with high dielectric constant to HSS/UHPC interface. Therefore, the charge transfer reaction is decreased and consequently the corrosion protection is enhanced.

## Conclusion

1. Corrosion resistance of HSS embedded in UHPC immersed in 3.5% NaCl in the absence and presence of 1% NS, NWG, NMK and NRHA is evaluated using open circuit potential, potentiodynamic polarization and electrochemical impedance spectroscopy techniques under normal and accelerated conditions.
2. Incorporation of the studied nano materials in UHPC, shifts both the E_ocp_ and E_corr_ of HSS to more noble values without any sign for the pitting corrosion, as well as decreases its I_corr_ compared to the control specimen in the normal and accelerated conditions.
3. EIS analysis illustrates that the presence of the studied nano materials enhances both the concrete bulk resistance and the charge transfer resistance at HSS/UHPC interface.
4. TGA analysis showed the order of the weight loss due to the dehydration of CSH is agreed with the order of the compressive strength and the corrosion resistance.
5. The inhibitory effect of the studied nano materials for the corrosion of HSS is interpreted on the basis of the change in the microstructure of UHPC that retards both the (1) diffusion of the corrosive ions and/or H_2_O/O_2_ molecules through the UHPC bulk to the HSS/UHPC interface, and (2) the flow of the electrons between the anodic and cathodic sites, thus impeding the propagation of the corrosion process.
